# An Overview of *Aegilops tauschii*-Associated Wheat Damage and Generic Resources

**DOI:** 10.3390/plants15182784

**Published:** 2026-09-11

**Authors:** Libing Yuan, Yaling Geng, Chencan Wang, Yan Cui, Xu Dong, Zongran Su, Linghui Wang

**Affiliations:** 1 Plant Protection Institute, Hebei Academy of Agriculture and Forestry Sciences, Baoding 071001, China; gengyaling2006@163.com (Y.G.); w3387134833@163.com (C.W.); dongxuzb@163.com (X.D.); 15097791534su@sina.com (Z.S.); wlh15932127411@163.com (L.W.); 2 National Collection of Plant-Associated Microbes (Hebei), Baoding 071001, China; 3 IPM Innovation Center of Hebei Province, Baoding 071001, China; 4 International Science and Technology Joint Research Center on IPM of Hebei Province, Baoding 071001, China; 5 Hebei Plant Protect and Plant Inspect Station, Shijiazhuang 050021, China; cuiyan182026@163.com

**Keywords:** *A. tauschii*, weed, *Triticum aestivum* L., integrated pest management, wheat breeding

## Abstract

*A. tauschii* is the wild progenitor that contributed the D genome to common wheat. Since the 1990s, however, it has progressively become a destructive weed in northern China, posing a serious threat to wheat production. This review systematically summarizes current knowledge of *A. tauschii*, including its taxonomy, population differentiation, mechanisms underlying infestation and crop damage, environmentally sustainable management strategies, and the exploitation of its genetic resources. Two ecotypes have been identified in China: the wild-type and the weed-type. The weed-type spreads rapidly through pathways such as contaminated seed lots. Owing to its highly synchronized life cycle with winter wheat, strong environmental adaptability, prolific reproductive capacity, and superior competitive ability for ecological niches, *A. tauschii* can rapidly establish populations within wheat fields, resulting in substantial yield losses. Current management strategies emphasize an integrated control system combining primary agronomic practices, chemical control for rapid intervention, and smart agricultural technologies to enhance monitoring and precision management. *A. tauschii* represents an invaluable reservoir of genes associated with abiotic stress tolerance, disease resistance, and yield-related traits. Future research should integrate advances across multiple disciplines to simultaneously improve weed management and maximize the utilization of this important genetic resource.

## 1. Introduction

*Aegilops tauschii* Coss. (*A. tauschii*), a member of the tribe Triticeae and one of the closest wild relatives of wheat, is of fundamental importance for understanding the origin and evolution of common wheat (*Triticum aestivum*). As the direct donor of the D genome to hexaploid wheat, *A. tauschii* occupies a unique position in wheat genetics and breeding. However, changes in agricultural production systems and agroecosystems have altered its ecological role. Since the 1990s, *A. tauschii* has increasingly emerged as a highly problematic weed throughout the Yellow River Basin and other major wheat-growing regions of northern China. Its rapid spread, difficulty of control, and severe competitive effects have made it a major constraint on stable wheat production. Large-scale outbreaks were first reported in China at the beginning of the 21st century, and the species has now been documented in more than 16 provinces and municipalities.

**Figure 1:**
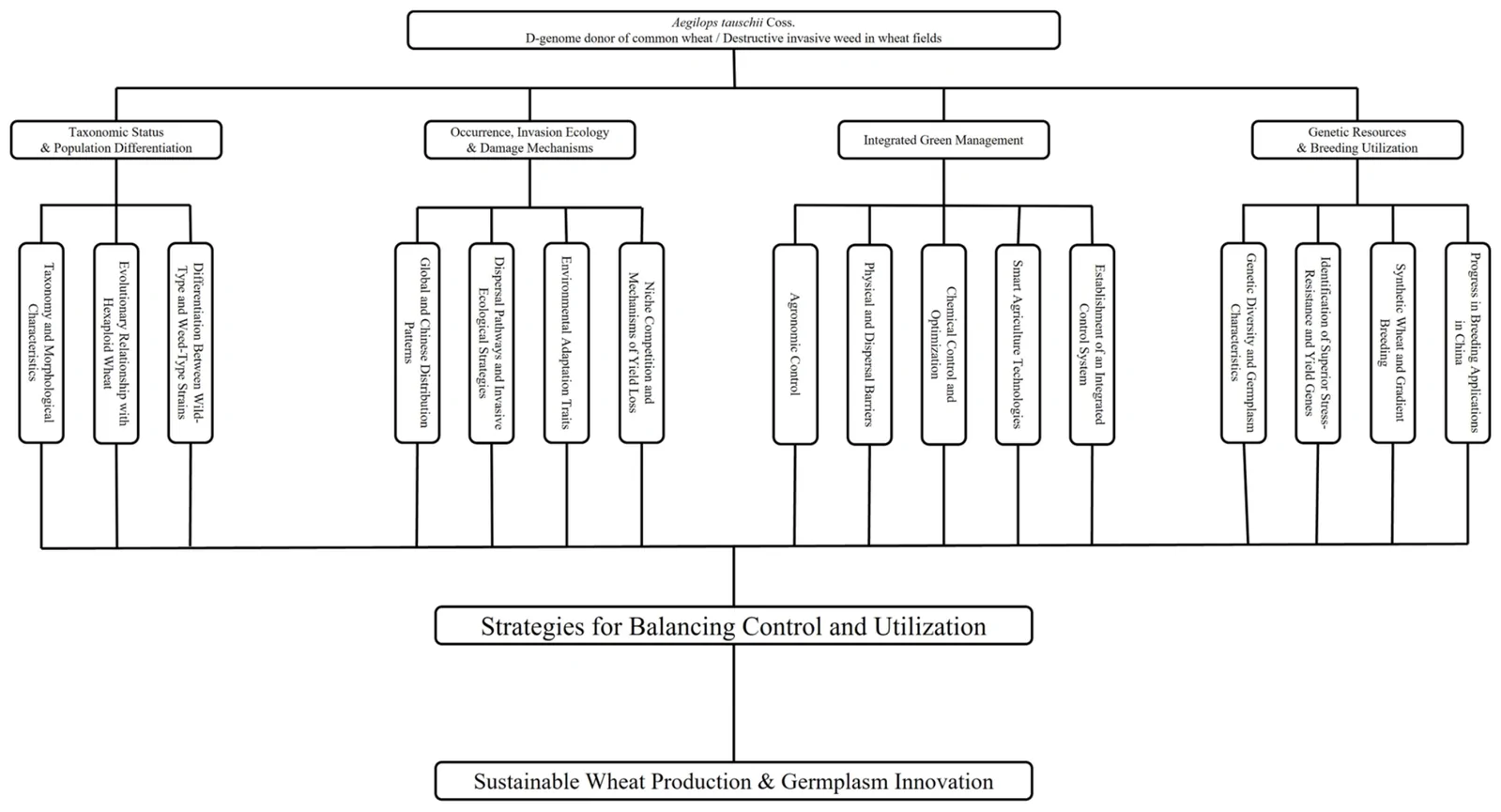
Conceptual framework illustrating the overall structure of the review and the dual roles of *A tauschii* in wheat production systems.

As an invasive and highly competitive weed of wheat fields, *A. tauschii* presents substantial challenges because of its biological characteristics, ecological adaptability, and efficient dispersal mechanisms. Consequently, there is an urgent need for a comprehensive understanding of its biology and management. This review synthesizes recent research on *A. tauschii*, with emphasis on its taxonomy and population differentiation, mechanisms of infestation and crop damage, management strategies, and the exploitation of its genetic resources (Figure 1). By integrating findings from multiple disciplines, this review aims to provide a comprehensive reference for both the integrated management of *A. tauschii* infestations and the effective utilization of its valuable genetic resources, thereby supporting the sustainable development of wheat production.

## 2. Taxonomic Status and Population Differentiation of *A. tauschii*

### 2.1. Taxonomy and Morphological Characteristics of A. tauschii

*A. tauschii* (2n = 2x = 14, DD), commonly referred to as Tausch’s goatgrass, belongs to the genus Aegilops L. within the tribe Triticeae of the family Gramineae (Poaceae). It is a diploid, predominantly self-pollinating species. Approximately 25 species have been described within the genus Aegilops, but *A. tauschii* is the only species reported to occur naturally in China [1], highlighting its distinctive taxonomic status. It is typically a winter annual herb that flowers and sets seed from April to June and reproduces exclusively by seed. The root system is fibrous, delicate, and easily damaged. Plants typically reach 30–80 cm in height, with culms arising in groups and frequently bent near the base. The leaf blades are approximately 0.5 cm wide, slightly rough to the touch, and sparsely pubescent on the adaxial surface. Leaf sheaths tightly clasp the stem and bear cilia along their margins. The terminal inflorescence is a spike composed of solitary spikelets embedded within depressions of the rachis; at maturity, the rachis disarticulates into individual segments. The fruit is a dull, dark yellowish-brown caryopsis. When ripe, it is tightly enclosed by adherent palea and lemma [2].

Based on morphological characteristics, *A. tauschii* is classified into two subspecies: *A. tauschii* ssp. *tauschii* and *A. tauschii* ssp. *strangulata* [3]. The subspecies *tauschii* consists of three recognized varieties: *A. tauschii* ssp. *tauschii* var. *meyeri*, var. *typica*, and var. *anathera*. The two subspecies differ markedly in spike morphology. Plants belonging to ssp. *tauschii* produce slender, cylindrical spikes, whereas those of ssp. *strangulata* are characterized by short, thick, bead-like spikes with glumes that are wider than long and a twisted rachis [4]. Molecular studies have further grouped *A. tauschii* into T (*tauschii*) and S (*strangulata*) genotypes [5], reflecting substantial genetic divergence within the species. Genome-wide association analyses assessed the correspondence between morphological variation and genetic background [5], providing an important framework for taxonomic identification and breeding applications.

### 2.2. Evolutionary Relationship with Hexaploid Wheat and Population Differentiation in China

*A. tauschii* is one of the wild progenitors of hexaploid common wheat (*Triticum aestivum*, AABBDD) and serves as the exclusive donor of the D genome. The hexaploid wheat genome originated through two successive rounds of natural hybridization followed by chromosome doubling, involving *Triticum urartu* (AA), *Aegilops speltoides* (BB), and *A. tauschii* (DD) [6,7,8,9,10]. Initially, hybridization between the AA and BB genomes generated tetraploid emmer wheat (*Triticum turgidum* subsp. *dicoccum*, AABB). This tetraploid ancestor subsequently hybridized with *A. tauschii*, and the resulting hybrid underwent spontaneous chromosome doubling, producing fertile hexaploid common wheat. The acquisition of the D genome not only completed the genomic composition of modern bread wheat but also substantially enhanced its tolerance to environmental stresses, resistance to a broad spectrum of diseases, and end-use quality, facilitating its worldwide cultivation as one of the three major staple food crops. Meanwhile, *A. tauschii* has retained much of the genetic diversity present in its wild ancestors and therefore represents an indispensable reservoir of novel alleles for overcoming the genetic limitations of modern wheat breeding programs. Multiple lines of molecular evidence support this evolutionary relationship. Cytogenetic studies have demonstrated a high degree of chromosome homology between the D genome of common wheat and that of *A. tauschii* [4], while molecular marker analyses and whole-genome sequencing have revealed greater than 99% sequence similarity between their D genomes [11]. Together, these findings provide compelling evidence that *A. tauschii* is the direct donor of the wheat D genome.

As the ancestral source of the D genome, *A. tauschii* has undergone substantial ecological differentiation during its long evolutionary history under both natural selection and human agricultural activities. This process has been particularly pronounced in China, where populations have adapted to contrasting natural and agricultural environments, resulting in the emergence of two distinct ecotypes: the wild-type and the weed-type.

Over its long evolutionary history, *A. tauschii* has undergone pronounced ecological differentiation driven by both natural selection and human agricultural activities. In China, this adaptive divergence has resulted in two distinct ecotypes occupying contrasting habitats: the wild-type and the weed-type. Wild-type populations are indigenous to natural steppe ecosystems, primarily distributed across the Yili River Valley of Xinjiang at elevations ranging from 600 to 1500 m. Cultivation trials and cytological analyses have verified their native status [12,13], extending the documented eastern limit of the species’ natural distribution to approximately 83.5° E. In contrast, weed-type populations are exclusively associated with agroecosystems, where they infest wheat, barley and other cereal crops. The earliest records of weedy *A. tauschii* in China date to 1955–1956 from the middle Yellow River basin. The evolutionary origin of these agricultural populations remains unresolved. One hypothesis posits an eastward range expansion from Xinjiang’s wild populations concurrent with early cereal cultivation, supported by cytogenetic evidence of genetic similarity between the two groups [13,14]. An alternative hypothesis, grounded in microsatellite molecular fingerprinting, suggests Chinese weedy populations are genetically more similar to Iranian wild accessions, and were likely introduced from West Asia via the Silk Road during the historical spread of wheat cultivation [15]. Additional proposed introduction routes include contaminated imported forage and international grain trade [16,17,18,19,20,21]. Clarifying the origin and dispersal history of weedy *A. tauschii* will require further integration of population genomics and historical biogeographic evidence.

## 3. Occurrence, Invasion Ecology and Damage Mechanisms of *A. tauschii*

### 3.1. Global Distribution Pattern and Geographic Occurrence of A. tauschii

**Figure 2:**
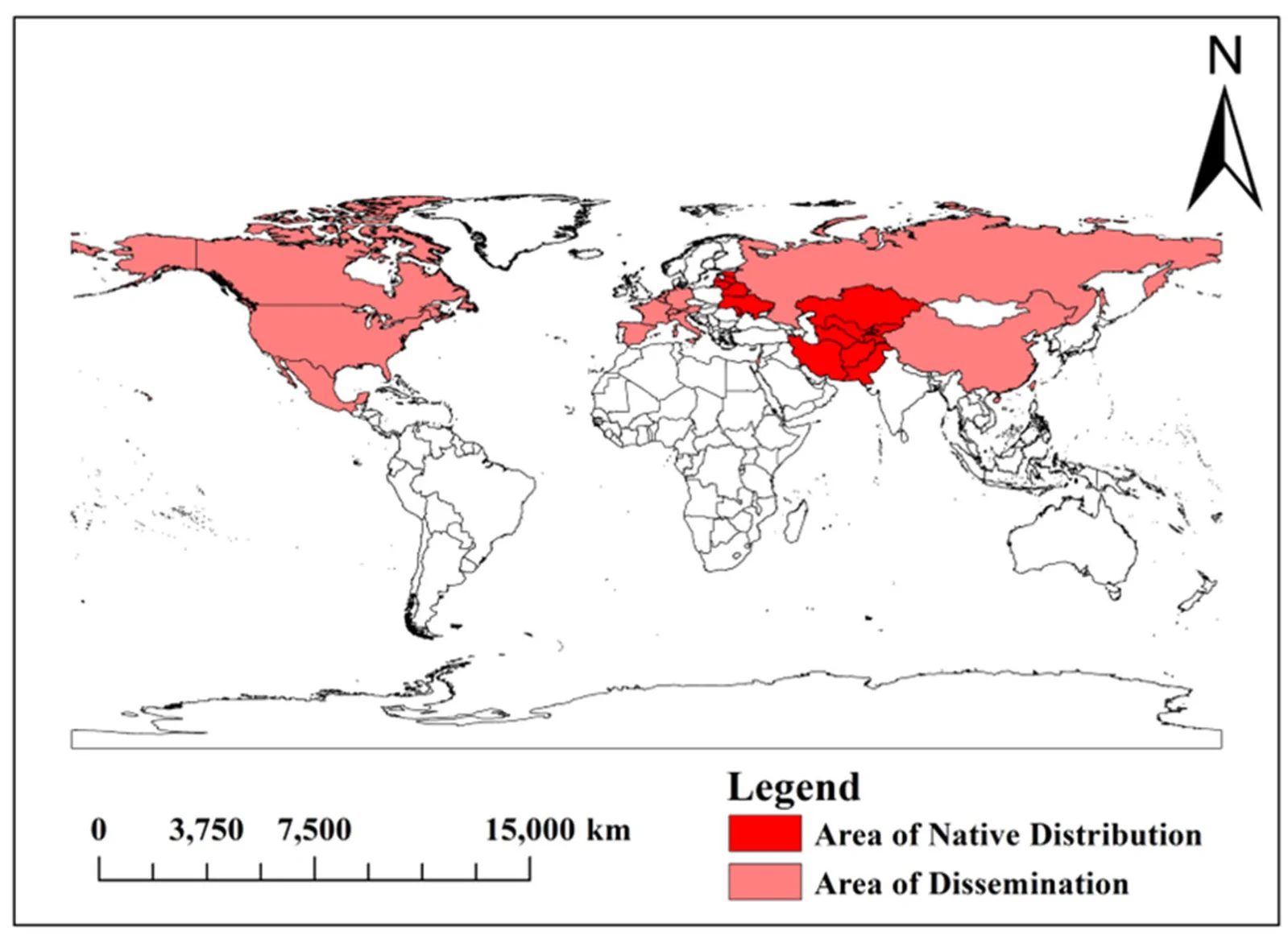
Geographical distribution of *A. tauschii* in the world. This map was constructed based on data from previous studies (References [12,22,23,24,25]).

The native distribution of *A. tauschii* is concentrated within a relatively narrow geographic region centered near 50° E longitude around the southern Caspian Sea and Azerbaijan. Its principal distribution extends across the Middle East, including Iran, Afghanistan, and Pakistan, and continues into Central Asia and parts of Eastern Europe (Figure 2) [22,23,24]. Iran is widely recognized as both the center of origin and the primary center of genetic diversity for the species [25]. Across its range, *A. tauschii* occurs in two principal ecological forms: natural populations inhabiting native ecosystems and weedy populations associated with cultivated land. In northern Iran and the Transcaucasus, the species is a characteristic component of steppe and forest-steppe vegetation. By contrast, in southern Iran, southern Afghanistan, and the Quetta region of Pakistan, it has successfully invaded agricultural land and become a dominant weed in wheat production systems (Figure 2) [12]. Using the MaxEnt ecological niche model, Fang et al. predicted that the global areas most suitable for *A. tauschii* are concentrated between 30° N and 45° N, encompassing major wheat-producing regions across West Asia, the Middle East, southeastern Europe, and the Mediterranean coast of North Africa [26]. This predicted distribution closely parallels the global distribution of wheat cultivation, providing highly favorable ecological conditions for the establishment and spread of the species as an agricultural weed.

*A. tauschii* is recognized as a troublesome wheat-field weed in parts of Europe and West Asia, yet comprehensive studies on its biology and management in these regions remain scarce. By contrast, its closely related congener *A. cylindrica* has been the subject of extensive systematic research as a major invasive wheat weed in the western United States [27,28,29,30,31,32,33]. By 1993, *A. cylindrica* had infested approximately 2 million hectares in the U.S., causing an estimated USD 145 million in economic losses. After nearly two decades of integrated management, infested areas across 14 western U.S. states declined by 45–55%, though imazamox-resistant populations have since been reported [34]. This successful management framework offers important insights for *A. tauschii* control in China, emphasizing diversified crop rotation to deplete the soil seed bank and long-term herbicide resistance monitoring to delay resistance evolution. Compared with the extensive and systematic research conducted on *A. cylindrica* in the United States, investigations of the closely related and economically important wheat weed *A. tauschii* in China began relatively late. Nevertheless, records of its occurrence date back to the 1950s, and its invasion history can be divided into distinct stages characterized by gradual establishment, local expansion, and rapid dissemination. The earliest quantitative assessment of weed-type *A. tauschii* damage was reported by Wang et al. [35], who conducted a systematic survey of wheat fields in the Weinan reclamation area of Shaanxi Province in 1982. Their investigation documented an average population density of 0.06 plants m^−2^, with a maximum density of 174 plants m^−2^. The corresponding relative density, occurrence frequency, relative frequency, and damage index were 0.28%, 0.0881%, 2.48%, and 2.76%, respectively. Based on these observations, the infestation was classified as Grade II, indicating widespread occurrence across most surveyed fields with appreciable impacts on wheat production. This study represents the first scientific documentation of *A. tauschii* as a damaging weed in China. During the following decade, however, infestations remained sporadic and generally caused limited economic losses, resulting in relatively few published reports.

**Figure 3:**
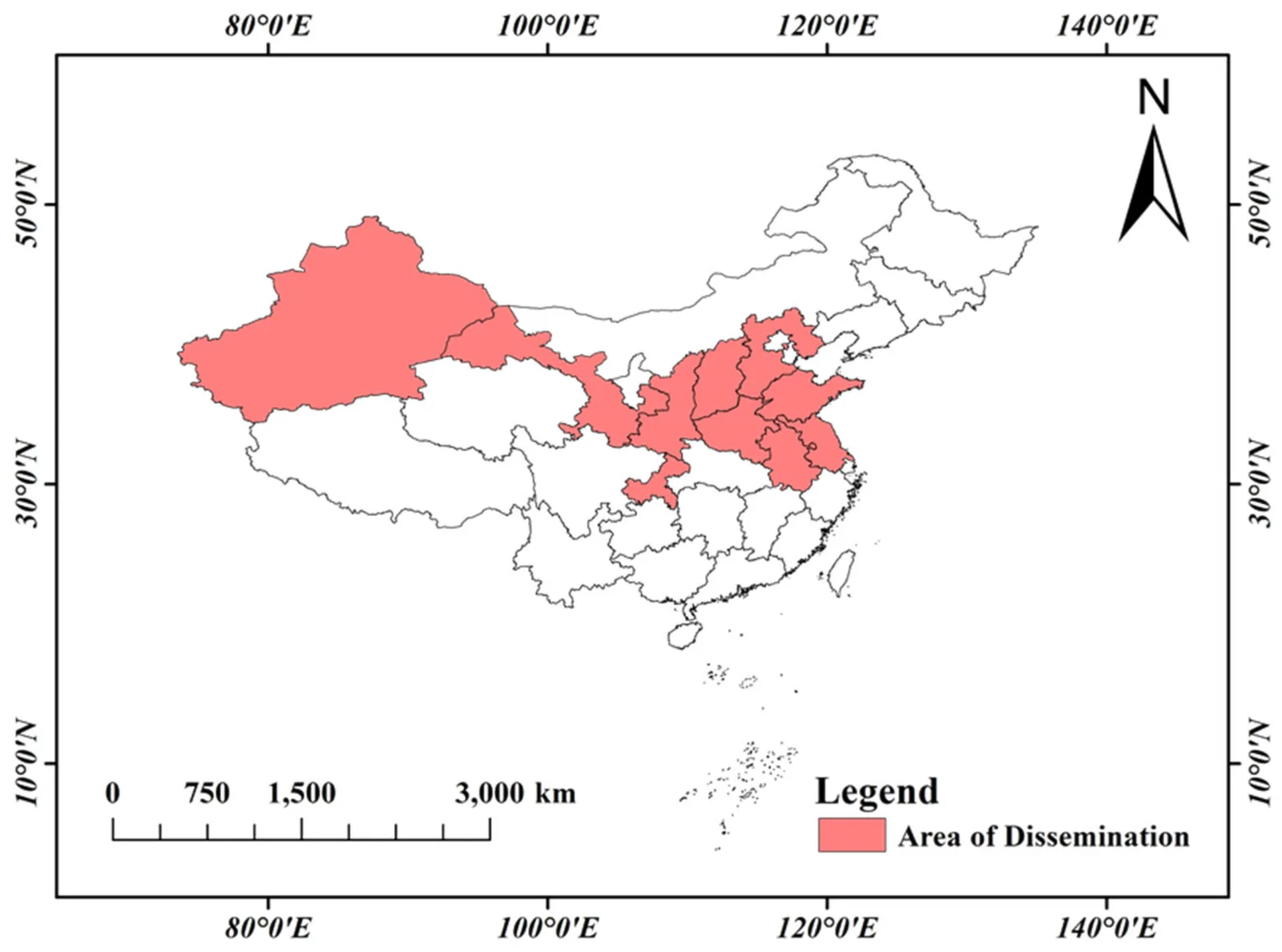
Geographical Distribution of *A. tauschii* Across Provinces in China (Data as of 2024). This map was constructed based on data from previous studies (References [20,35,36,37,38,39,40,41,42,43,44,45]).

Beginning in the 1990s, *A. tauschii* became increasingly evident in several agricultural regions. In 1992, the Henan Provincial Collaborative Research Group on Integrated Pest Management for Watermelon Diseases, Pests, and Weeds [36] recorded *A. tauschii* among 180 weed species representing 38 families in watermelon production systems, suggesting that the species had expanded beyond wheat fields into other cultivated crops. In 1995, Liu et al. [37] documented increasing infestations in floodplain wheat fields in Weinan, Shaanxi Province, where severe infestations caused substantial yield reductions in some fields. During the same period, *A. tauschii* spread into Fengxian County, Jiangsu Province, where it rapidly developed into a major weed in local wheat production and subsequently expanded into neighboring counties and cities [38]. In 1998, Shang [39] reported its occurrence in wheat fields in Kaifeng, Henan Province, although the infestation remained relatively minor. One year later, Hao [40] included difenzoquat as a recommended herbicide for controlling *A. tauschii* and other grassy weeds in the handbook Use Techniques for Wheat Field Herbicides. However, the publication provided little information regarding the species’ distribution or population dynamics, reflecting the limited understanding of its biology and invasion status at that time (Figure 3). Overall, although *A. tauschii* had been detected in multiple regions during the 1990s, its distribution remained localized, and the extent of damage was still relatively limited.

The severity and geographic extent of *A. tauschii* infestations increased dramatically after the beginning of the 21st century. In 2000, Zhang et al. [41] reported a serious infestation in a 1.33-ha wheat field in Qianchang Village, Yanshan County, Hebei Province, where the species had first been observed in 1999. Infestation rates ranged from 5% to 10%, with localized areas reaching 40%, resulting in severe suppression of wheat growth. As infestations expanded both geographically and in intensity, the number of related investigations and reports increased substantially [37,42,43]. Before 2000, confirmed infestations had been restricted to Shaanxi, Henan, and Hebei Provinces. By 2007, however, Zhang et al. [20] reported that the weed had spread to eight provinces, including Shanxi, Shandong, Chongqing, Inner Mongolia, and Jiangsu, affecting approximately 330,000 ha of wheat fields and exhibiting a clear trend of rapid geographic expansion accompanied by increasing damage. More recent reports indicate that *A. tauschii* has now spread to Beijing, Tianjin, Anhui, Hubei, Xinjiang, Gansu, Sichuan, and Guangdong, bringing the total number of affected provinces and municipalities to 16 [44,45]. Its potential distribution now encompasses nearly all major winter wheat production regions in China (Figure 3).

From its initial incidental detection during the 1950s, through the first documented damage assessment in the 1980s, to its widespread expansion during the 21st century, *A. tauschii* has evolved into one of the most important invasive weeds threatening wheat production and agroecosystem stability in China. Consequently, continued research into its biology, ecology, and integrated management remains a high priority.

### 3.2. Dispersal Pathways and Invasion Ecology

The increasing impact of *A. tauschii* on wheat production has attracted considerable attention from both researchers and agricultural practitioners. The continuing expansion of its geographic distribution is primarily driven by its highly efficient dispersal capacity, which results from the combined effects of human activities and natural processes. Seed movement represents the dominant mechanism underlying its spread. Multiple pathways contribute to seed dispersal, including contamination during seed production and distribution, transport by agricultural machinery, movement through irrigation systems, and introduction via organic fertilizers. Because *A. tauschii* seeds closely resemble wheat seeds in both size and morphology, they can easily contaminate commercial seed lots and be transported over long distances during seed production and marketing, thereby facilitating interregional spread [46,47]. The potential for international dissemination has also been demonstrated by repeated interceptions of *A. tauschii* seeds at Chinese ports in imported commodities originating from countries such as France and India. Agricultural machinery constitutes another important dispersal vector, particularly at the local scale. During harvesting and field operations, combines and other farm equipment readily accumulate mature *A. tauschii* seeds on machinery surfaces, tracks, and working components. When this equipment is subsequently used in other fields, the attached seeds are dispersed, accelerating the spread of the weed among wheat production areas [48,49]. Natural processes further contribute to local dispersal. Irrigation runoff can transport seeds across and between fields, while incompletely decomposed organic fertilizers containing viable seeds may introduce new infestations during fertilizer application, thereby promoting continued local establishment and population expansion.

From the perspective of invasion ecology, the remarkable success of *A. tauschii* in wheat agroecosystems can be attributed to a combination of ecological strategies that incorporate characteristics of both r-selected and K-selected species [50,51]. On one hand, the species displays typical r-strategy traits, including prolific seed production, pronounced seed dormancy, and the ability to establish persistent soil seed banks following seed dispersal. On the other hand, *A. tauschii* also exhibits strong competitive ability characteristic of K-selected species, enabling it to compete effectively with wheat for light, water, nutrients, and ecological space, thereby reinforcing its dominance within invaded fields. Meanwhile, widespread adoption of conservation tillage and other modern agricultural practices has altered farmland ecosystems, driving continuous shifts in weed community composition. These changes have favored the proliferation of highly competitive weed species, allowing *A. tauschii* to become increasingly dominant in many wheat-growing regions. Predictions generated using the MaxEnt ecological niche model [52,53] further indicate that the species has not yet occupied its full potential distribution. High-risk areas for future invasion include Chongqing, western Sichuan, Hubei, Anhui, Henan, the northern Guanzhong Plain of Shaanxi, Hainan, northern and western Xinjiang, and western Tibet. These projections provide valuable guidance for surveillance and early-warning programs. Accordingly, effective management will require integration of ecological principles with agronomic practices, supported by continuous monitoring to protect agricultural ecosystems and biodiversity while limiting further expansion of *A. tauschii*. In addition, investigating the responses of *A. tauschii* to fluctuations in resource availability within farmland ecosystems and elucidating its competitive interactions with wheat will improve our understanding of its invasion mechanisms and provide a stronger theoretical foundation for developing more effective ecological regulation and integrated management strategies.

### 3.3. Environmental Adaptability of A. tauschii

The exceptional invasive capacity of *A. tauschii* in winter wheat production systems is largely attributable to its distinctive germination and seedling emergence characteristics. Studies have shown that its seeds mature in late May and gradually lose dormancy during storage. After approximately 4.5 months, corresponding to early October, dormancy is almost completely released and seedling emergence reaches its peak. This timing closely matches the sowing season for winter wheat, allowing *A. tauschii* to exploit the same ecological niche as the crop from the earliest stages of establishment and thereby gain a pronounced competitive advantage.

Seed germination and emergence are jointly regulated by dormancy status and environmental conditions [54]. Under ambient storage conditions, dormancy release follows a predictable sequence. Germination begins to increase approximately seven weeks after seed maturation, rises rapidly between weeks 7 and 13, slows during weeks 14–20, and stabilizes after approximately 20 weeks. Temperature is one of the principal determinants of germination behavior. Optimal germination occurs between 15 and 20 °C, whereas the most rapid and uniform seedling emergence occurs at approximately 20 °C. In contrast, temperatures exceeding 25 °C or falling below 5 °C markedly suppress both germination and emergence [54,55,56].

Beyond temperature, environmental moisture, soil pH, and salinity also strongly influence the establishment of *A. tauschii*, highlighting its broad ecological adaptability [55]. Under moderate water stress, corresponding to soil osmotic potentials between −0.4 and −0.1 MPa, both germination and seedling emergence are significantly enhanced, with emergence showing the greater response. Even when osmotic potential declines to −0.6 MPa, seed germination remains largely unaffected despite a reduction in seedling emergence. These findings suggest that *A. tauschii* possesses substantial tolerance to low water availability, enabling successful establishment under relatively dry or poorly drained conditions and potentially providing a competitive advantage over other weed species experiencing water stress [56]. The species also exhibits broad pH tolerance, with seeds capable of germinating across a wide range of soil pH values, an important characteristic supporting its extensive ecological distribution [57]. Salinity exerts a progressively inhibitory effect on both germination and emergence as salt concentrations increase. Nevertheless, *A. tauschii* demonstrates moderate salt tolerance overall, allowing it to maintain competitive performance in moderately saline environments. This observation is consistent with reports by Hun et al. describing severe infestations of *A. tauschii* in saline–alkaline wheat fields in Cangzhou, Hebei Province [58,59,60,61] (Table 1).

The biological characteristics of *A. tauschii*, including predictable dormancy release within approximately 4.5 months, shallow emergence synchronized with winter wheat planting, broad tolerance to temperature and soil pH, and moderate resistance to water deficit and salinity, provide a strong ecological foundation for its rapid establishment and spread in wheat fields. Although extreme drought and high salinity can restrict seedling establishment, these limitations do little to diminish its overall invasive potential. Future research should therefore focus on elucidating the physiological and molecular mechanisms regulating seed dormancy and germination to facilitate the development of more targeted and effective management strategies.

### 3.4. Damage Mechanisms: Niche Competition and Yield Loss

The severe impact of *A. tauschii* on wheat production stems primarily from the extensive overlap in ecological niches shared by the weed and its host crop, resulting in intense competition for essential resources throughout the growing season. In terms of light interception, *A. tauschii* exhibits vigorous tillering and frequently exceeds wheat in plant height, enabling it to develop dense canopies that shade neighboring wheat plants. This shading reduces the interception of photosynthetically active radiation, decreases canopy photosynthetic efficiency, and ultimately limits dry matter accumulation and assimilate translocation in wheat. Below ground, the species possesses a vigorous and deeply penetrating root system capable of efficiently exploiting soil moisture and nutrients, particularly nitrogen, phosphorus, and potassium. Competition for these resources weakens wheat growth and further compromises crop productivity. Dense infestations also occupy valuable aboveground and belowground space, suppressing tiller formation, reducing the number of productive spikes, and disrupting the structural stability of wheat populations [60].

Following introduction into a new area, *A. tauschii* typically begins with extremely small founder populations but is capable of remarkably rapid population expansion. Long-term field experiments have demonstrated that, in the absence of artificial control measures, initial densities ranging from 1 to 60 plants m^−2^ can establish persistent populations that increase dramatically within only a few growing seasons. When initial densities are as low as 1–2 plants m^−2^, population size may increase by approximately 190-fold by the second growing season. Initial densities of 4–10 plants m^−2^ can increase roughly 60-fold during the following year, while even populations beginning at 60 plants m^−2^ are capable of expanding by approximately 34-fold. These observations illustrate the exceptional colonization ability and environmental adaptability of *A. tauschii*, as well as the often inconspicuous nature of early infestations. Although the impact on wheat may be limited during the initial invasion stage, continued population growth can result in yield reductions ranging from 14% to 52% by the second growing season [49].

Plant biomass provides an effective indicator of competitive intensity between species. As *A. tauschii* density increases, its suppressive effect on wheat becomes progressively more severe. This competitive pressure primarily reduces the number of productive wheat spikes and decreases thousand-kernel weight, leading to substantial declines in both grain yield and quality. Field studies have consistently demonstrated a strong density-dependent relationship between *A. tauschii* infestation and crop loss. Infestation levels of 1–50 plants m^−2^ are associated with yield reductions of approximately 5–10%, whereas densities exceeding 100 plants m^−2^ can cause yield losses greater than 50%, with complete crop failure reported under extreme infestation conditions [21,63,64,65,66,67,68]. Beyond direct reductions in productivity, contamination of harvested grain with *A. tauschii* seeds lowers varietal purity and commercial grain quality, thereby diminishing processing value and marketability. Moreover, *A. tauschii* can function as an alternate or intermediate host for numerous insect pests and plant pathogens, increasing inoculum and pest reservoirs within production systems and amplifying long-term biotic stress on wheat. Consequently, preventing the spread of *A. tauschii* into previously uninfested areas should remain a priority. In fields where infestations are already established, prompt intervention and early control are essential to minimize the initial population size and suppress subsequent population expansion and associated crop losses.

## 4. Green Prevention and Control Technology for *A. tauschii*

The invasion of *A. tauschii* is characterized by its cryptic establishment, long-term persistence, and strong dispersal capacity. Effective and sustainable suppression therefore requires an integrated management approach that combines multiple complementary control strategies rather than relying on any single measure.

### 4.1. Agronomic Cultural Control Measures

Agronomic practices constitute the cornerstone of environmentally sustainable management of *A. tauschii*. By optimizing cropping systems and field management, production environments can be modified to discourage weed establishment while simultaneously promoting vigorous wheat growth, thereby reducing the likelihood of population expansion and economic damage. Contaminated seed is one of the principal pathways responsible for the long-distance dissemination of *A. tauschii*. Consequently, rigorous seed cleaning should be incorporated into wheat production systems. Physical separation techniques, including winnowing and mechanical screening, should be used to remove *A. tauschii* seeds from commercial wheat seed lots, thereby interrupting seed-mediated dispersal at its source [46,49]. Crop rotation is another highly effective cultural practice because it disrupts the life cycle of *A. tauschii* by altering the ecological conditions that favor its establishment. Under conventional winter wheat-summer maize double-cropping system, particularly when combined with no-tillage and residue retention, large numbers of *A. tauschii* seeds accumulate on the soil surface each year, progressively increasing infestation severity. Replacing this system with two-year, three-crop rotations, such as winter wheat–summer maize–spring soybean, winter wheat–summer maize–spring peanut, or winter wheat–summer maize–spring corn, substantially alters soil conditions and weed emergence dynamics, resulting in significantly lower *A. tauschii* populations than those observed under continuous double cropping. Field trials in Hebei Province further confirmed that these three rotation patterns reduced weed density from 186 stems m^−2^ to 11–15 stems m^−2^ after one rotation cycle, demonstrating robust and durable control efficacy [62]. Likewise, rotating wheat with broadleaf crops such as oilseed rape or cotton for two to three years effectively modifies the weed community and suppresses further population expansion [48,65].

The emergence of *A. tauschii* is also strongly influenced by seed burial depth. Seedlings emerge most successfully from soil depths of 1–5 cm, whereas emergence declines sharply when seeds are buried below 5 cm and is completely prevented at depths greater than 15 cm. Independent studies on multiple Chinese populations have consistently verified this pattern: Zhang et al. [69] reported no seedling emergence at a burial depth of 6 cm, with a 50% reduction in maximum emergence occurring at approximately 2.09 cm; Wang et al. [70] further demonstrated that emergence was fully inhibited at depths exceeding 8 cm across three geographically distinct populations. Successful emergence depends on nutrient reserves stored in the endosperm, and seedlings originating from excessive depths are unable to elongate sufficiently to reach the soil surface. Instead, deeply buried seeds often remain dormant and contribute to a persistent soil seed bank [61]. Consequently, conventional shallow rotary tillage, which disturbs only the upper soil layer, inadvertently favors synchronized emergence by concentrating seeds within their optimal germination zone. In contrast, deep plowing to depths of at least 15 cm buries many surface seeds below their emergence threshold. These seeds are subsequently unable to establish because of physical limitations, reduced oxygen availability, and depletion of endosperm reserves. Field studies have consistently demonstrated a significant negative relationship between plowing depth and *A. tauschii* infestation, confirming the effectiveness of deep tillage in reducing field emergence [66,71,72,73,74].

In addition, maize straw residue mulch on the soil surface can linearly reduce seedling emergence of *A. tauschii* by creating physical obstruction and altering the soil microenvironment. Wang et al. [72] reported that seedling emergence decreased from above 89% with no residue to 64–76% at a residue application rate of 15 t ha^−1^, providing a potential non-chemical control option suitable for straw-retention farming systems. Appropriate irrigation, fertilization, and planting density can further enhance wheat competitiveness and suppress weed growth through intensified crop competition. In practice, pre-sowing irrigation may stimulate early germination of *A. tauschii* seeds, after which rotary tillage can destroy the emerging seedlings and reduce the soil seed bank. Field trials showed that inducing weed emergence via irrigation 15 days before wheat sowing, followed by rotary tillage, reduced *A. tauschii* density by 74% before wintering and 63% after spring regrowth compared with untreated plots [62]. Because the optimum germination temperature for *A. tauschii* ranges from 15–20 °C, moderately delaying wheat sowing may expose germinating weeds to rapidly declining autumn temperatures, shortening their growth period and reducing successful establishment. However, sowing should not be postponed excessively, as delayed planting may also compromise wheat development. This effect has been quantitatively validated: later sowing dates significantly reduced weed density in the field, although excessive delays may also compromise wheat development [62]. Similarly, appropriate seeding rates and balanced fertilizer management improve wheat vigor and canopy competitiveness, thereby suppressing *A. tauschii* through enhanced crop competition [42,62].

Manual removal also requires careful management. If uprooted *A. tauschii* plants are left in the field, mature seeds may disperse and replenish the soil seed bank, resulting in greater infestations during subsequent growing seasons. Therefore, all removed plants should be promptly collected and destroyed outside the field to eliminate this source of seed production.

### 4.2. Physical Control and Transmission Blocking Measures

Physical control and measures that interrupt dispersal pathways complement agronomic management by directly eliminating existing plants and preventing the movement of seeds into new areas. These approaches are particularly valuable for preventing establishment and containing localized infestations. Plant quarantine represents the primary defense against long-distance dispersal of *A. tauschii*. Strict quarantine procedures should be implemented throughout both seed production and seed transportation. In particular, movement of wheat seed originating from infested areas into regions free of *A. tauschii* should be prohibited to prevent regional expansion of the weed [46,49]. Manual removal remains the most practical and effective strategy where infestations are sparse or newly established. Eradication efforts should be conducted during the early vegetative stage, when plants are relatively small and readily distinguished from wheat. Eliminating isolated plants and small patches before seed production can effectively prevent local population establishment and subsequent spread. Human activities play a major role in the short-distance dispersal of *A. tauschii*. Accordingly, farmer education should be strengthened through technical training programs and extension materials that improve species identification and promote scientifically based management practices. Particular emphasis should be placed on seed cleaning, machinery sanitation, and maintaining field hygiene to minimize inadvertent human-mediated dispersal [21,44].

### 4.3. Chemical Control: Application and Limitations of Mesosulfuron-Methyl

Chemical herbicides remain the most rapid and effective means of suppressing *A. tauschii* infestations. Because *A. tauschii* is closely related to wheat and occupies a highly similar ecological niche, however, selective herbicide options are extremely limited. At present, mesosulfuron-methyl is the only post-emergence herbicide that consistently provides high efficacy against *A. tauschii* in winter wheat production systems in China. A systematic herbicide screening trial confirmed that mesosulfuron-methyl reduced weed fresh biomass by 93%, significantly outperforming pyroxsulam (28% reduction), flucarbazone-sodium (7% reduction), fenoxaprop-P-ethyl (5% reduction) and clodinafop-propargyl (3% reduction) [62]. Multiple independent herbicide evaluation studies have further corroborated this conclusion. Li et al. [75] tested four commonly used wheat-field herbicides against *A. tauschii*, showing that mesosulfuron-methyl achieved approximately 80% fresh weight control efficacy, while flucarbazone-sodium, isoproturon, and haloxyfop-P-methyl exerted only slight inhibitory effects, with the highest fresh weight control rate reaching only 34.22%, offering limited practical value for field production. Xu et al. [76] evaluated six herbicides for *A. tauschii* control, and found that mesosulfuron-methyl delivered 84.5% fresh weight control efficacy at the recommended field application rate, whereas the efficacy of diquat, flucarbazone-sodium, metolachlor, acifluorfen and isoproturon ranged from 15.2% to 47.1%. Furthermore, three consecutive years of multi-site field trials by Li et al. [38] consistently confirmed the reliable field efficacy of mesosulfuron-methyl against *A. tauschii*.

Mesosulfuron-methyl acts by inhibiting acetolactate synthase (ALS), thereby disrupting branched-chain amino acid biosynthesis and ultimately causing plant death [35,75]. Despite its effectiveness, mesosulfuron-methyl has several important limitations in practical production. First, crop safety remains a concern. Under adverse environmental conditions, particularly low temperatures or waterlogged soils, wheat is susceptible to herbicide injury. Even when the foliar safener mefenpyr-diethyl is applied, phytotoxicity has been reported in 10–32% of treated plants [77,78,79]. Second, the application window is relatively narrow. Maximum efficacy is achieved only when applications are made during the three- to six-leaf stage of wheat, whereas delayed treatment substantially reduces weed control [80]. Third, repeated and prolonged use creates strong selection pressure for herbicide resistance in *A. tauschii*, resulting in gradual declines in field efficacy. Furthermore, foliar application of mefenpyr-diethyl can increase herbicide tolerance not only in wheat but also in *A. tauschii*, thereby increasing the potential for inappropriate herbicide use and accelerating resistance evolution. Recent studies suggest that seed treatment with mefenpyr-diethyl offers an effective strategy for improving the safety profile of mesosulfuron-methyl. Seed-applied mefenpyr-diethyl selectively enhances wheat tolerance without increasing herbicide resistance in *A. tauschii*. Specifically, this treatment increases the wheat GR_50_ to mesosulfuron-methyl by 21.80-fold while leaving weed susceptibility unchanged. In addition, it accelerates herbicide metabolism within wheat, reducing the half-life of mesosulfuron-methyl by 46.87% compared with foliar application alone. At the physiological level, seed treatment alleviates inhibition of wheat ALS activity while increasing the activities of detoxification enzymes, including CYP450 and GST. At the molecular level, it induces the expression of multiple detoxification-related genes, including CYP450, GST, UGTs, and ABCs, through regulatory pathways involving transcription factors such as *AP2/ERF-ERF*, *bHLH*, *NAC*, and *MYB*, together with RLK-Pelle_DLSV protein kinases, thereby achieving coordinated responses that provide selective protection for wheat [81].

### 4.4. Smart Agriculture Control Technologies

Advances in smart agriculture have created new opportunities for the precise, efficient, and data-driven management of *A. tauschii*. By integrating digital technologies with conventional weed management practices, control programs can be implemented with greater accuracy while minimizing environmental impacts. These approaches improve both the scientific basis of management decisions and the efficiency of field operations, allowing effective weed suppression to be achieved alongside ecological sustainability. Modern remote sensing platforms enable rapid, real-time monitoring of *A. tauschii* infestations across large agricultural areas. Such technologies can accurately identify infestation hotspots, quantify their spatial extent, and assess damage severity, thereby providing reliable information for timely management decisions [82]. Coupling these monitoring systems with predictive modeling further enhances decision-making by forecasting population dynamics and potential spread, allowing preventative rather than reactive control strategies. For example, the MaxEnt ecological niche model developed by Fang et al. has been successfully applied to evaluate the invasion risk of *A. tauschii*, providing both theoretical support and practical guidance for regional management planning [46]. Information generated through remote sensing and predictive models can also support variable-rate herbicide application. Precision spraying based on infestation maps enables herbicides to be applied only where needed and at appropriate rates, substantially reducing chemical inputs while maintaining effective weed control. This targeted approach lowers production costs, decreases environmental contamination, and improves the balance between weed management efficacy and ecological protection [46].

### 4.5. Construction of an Integrated Control System

Long-term management of *A. tauschii* cannot be achieved through any single control strategy. Instead, sustainable management requires the establishment of a comprehensive, region-specific integrated management system that accounts for local ecological conditions and infestation severity. Such a framework should be founded on agronomic and cultural practices, reinforced by physical control and measures that interrupt dispersal, supported by chemical intervention when necessary, and strengthened through the application of smart agricultural technologies. Integrating multiple complementary practices, including plant quarantine, manual eradication, crop rotation, deep plowing, appropriately delayed wheat sowing, judicious herbicide application, and intelligent monitoring, offers the greatest potential for durable suppression of *A. tauschii* populations [83]. Practical implementation has already demonstrated the effectiveness of this integrated approach. In heavily infested areas of Hebei Province, combining deep plowing with crop rotation reduced field populations of *A. tauschii* by more than 30%. Similarly, in Jiangsu Province, an integrated management program incorporating ecological regulation, agronomic practices, and chemical control reduced field infestation densities by approximately 50% after three years, confirming both the practicality and long-term effectiveness of integrated management strategies [43]. Future research should prioritize long-term monitoring of *A. tauschii* population dynamics through permanent observation sites while developing molecular-assisted early warning systems capable of detecting emerging infestations before widespread establishment. Surveillance of herbicide-resistant populations should also be strengthened, together with the development of herbicides possessing novel modes of action and scientifically designed herbicide rotation programs to delay resistance evolution. In parallel, greater emphasis should be placed on identifying and utilizing wheat germplasm with enhanced resistance to *A. tauschii* and on developing biological management strategies, including the use of cover crops and natural enemies. Ultimately, interdisciplinary collaboration integrating weed science, agronomy, molecular biology, ecology, and digital agriculture will be essential for developing precise, intelligent, and regionally adapted integrated management systems capable of protecting both wheat production and agricultural ecosystems in China.

The integrated management system described above provides a practical framework for suppressing weedy *A. tauschii* populations and safeguarding wheat production. Notably, beyond its role as a problematic agricultural weed, *A. tauschii* also represents an irreplaceable genetic resource for wheat improvement. Reconciling effective weed control with germplasm conservation and utilization is a core principle for the sustainable management of this species.

## 5. Genetic Resources of *A. tauschii* and Gene Discovery

*A. tauschii* (DD), the direct diploid progenitor of the D genome of hexaploid bread wheat (Triticum aestivum, AABBDD), represents one of the most valuable wild relatives available for wheat genetic improvement because of its exceptional genetic diversity and broad-spectrum stress tolerance [84,85,86]. The remarkable diversity found within *A. tauschii* has arisen through the combined influences of evolutionary history, geographic isolation, and extensive genome plasticity. Originating in the Fertile Crescent, the species subsequently dispersed throughout Eurasia, where adaptation to diverse environments gave rise to genetically distinct ecotypes across regions such as the Iranian Plateau, the Central Asian steppes, and the arid landscapes of northwestern China. Geographic barriers, including mountain ranges and deserts, have restricted gene flow among populations and promoted further genetic divergence. For example, the Xinjiang population exhibits a Nei’s gene diversity index of 0.32, which exceeds the value reported for Iranian populations (0.28) [84]. The relatively high genetic diversity of Xinjiang populations may be attributed to long-term geographic isolation and distinct local adaptive evolution, forming a unique evolutionary lineage independent of the central diversity region. Moreover, more than 80% of the DD genome consists of transposable elements, generating extensive structural genomic variation. Concurrent expansion of stress-responsive gene families, including NBS-LRR and DREB, has further enriched its genetic repertoire and provides an important molecular basis for identifying elite alleles associated with stress adaptation [87].

*A. tauschii* possesses outstanding resistance to a broad spectrum of both abiotic and biotic stresses [88]. Under drought conditions, tolerance is achieved through physiological adjustments including proline accumulation and increased activities of antioxidant enzymes such as superoxide dismutase (SOD) and peroxidase (POD), both during water deficit and following rehydration [89,90,91]. Salt tolerance is mediated largely through the activity of the SOS1 gene, which facilitates Na^+^ compartmentalization, maintains intracellular ion homeostasis, and preserves a K^+^/Na^+^ ratio greater than 2:1. Osmotic adjustment through the accumulation of compatible solutes, including proline and glycine betaine, further contributes to adaptation under saline conditions, with reported salt tolerance thresholds of ECe = 12–18 dS/m [92,93]. Adaptation to low temperatures involves coordinated regulation by the Vrn-D1 vernalization gene together with the cold-responsive COR15A gene [94]. In addition to abiotic stress tolerance, *A. tauschii* exhibits strong resistance to numerous pathogens, including powdery mildew [95,96,97], stripe rust [98,99], and several other important pests and diseases affecting wheat [100,101]. Collectively, available evidence indicates that increasing geographic differentiation among *A. tauschii* populations is positively associated with enhanced stress tolerance, while expansion of stress-related gene families and extensive genomic structural variation provide key molecular mechanisms underlying these broad-spectrum adaptive traits.

The genetic diversity of *A. tauschii* substantially exceeds that of cultivated wheat, whose genetic base has been progressively narrowed through domestication and modern breeding [11]. Consequently, *A. tauschii* serves as an invaluable reservoir of novel alleles for improving wheat performance. Numerous genes and loci associated with disease resistance, abiotic stress tolerance, and yield-related traits have already been identified within its genome. Representative examples include the leaf rust resistance gene *Lr39* and the stem rust resistance gene *SrTA1662* (*Sr66*) [102]. Additional studies have identified drought-responsive transcription factors and disease resistance (R) genes that represent valuable genetic resources for breeding wheat cultivars with improved resilience to environmental and biological stresses [89,95]. Furthermore, the integration of high-density genetic linkage mapping with multi-environment phenotypic evaluation has enabled the identification of multiple stable quantitative trait loci (QTLs) influencing yield-related characteristics. Many of the favorable alleles at these loci originate from *A. tauschii*, and several species-specific haplotypes have been shown to positively regulate wheat grain length [103]. These characteristics establish *A. tauschii* as an indispensable germplasm resource for broadening the genetic diversity of cultivated wheat and accelerating the development of high-yielding, climate-resilient, and disease-resistant cultivars.

Advances in molecular biology and genomics have greatly accelerated the identification and utilization of elite genetic resources from *A. tauschii*. Early studies by Dvořák et al. established gene pool maps and clarified the genealogical relationships of *A. tauschii*, providing a theoretical framework for gene mapping and the selection of breeding materials [5,23]. Subsequently, Lagudah et al. demonstrated the high degree of homology between the D genome of *A. tauschii* and that of common wheat using molecular marker analyses [10]. Through genome-wide association studies (GWAS), Wang Lang identified several elite loci associated with low-phosphorus tolerance, providing valuable candidate genes for improving phosphorus-use efficiency in wheat [4]. Distant hybridization and the development of synthetic hexaploid wheat (SHW) have become major strategies for exploiting the genetic potential of *A. tauschii* [104]. Crosses between hexaploid and tetraploid wheat generate pentaploid hybrids, enabling D-genome chromatin from *A. tauschii* to be introgressed into the A and B genomes of tetraploid wheat and thereby creating abundant novel genetic variation [105]. Notably, the recently published chromosome-level genome assembly of the SHW-derived cultivar Chuanmai 104 provides a high-quality genomic reference for precise mining and utilization of *A. tauschii* genetic resources [106]. Introgression of favorable alleles from *A. tauschii* has been shown to substantially improve agronomic performance in wheat. For example, Olson et al. transferred the stem rust resistance gene *SrTA10187* from *A. tauschii* into wheat, significantly enhancing resistance to stem rust [107]. Using *A. tauschii*-wheat chromosome substitution lines, Huang identified multiple quantitative trait loci (QTL) controlling important agronomic traits, providing valuable resources for marker-assisted breeding [108]. Similarly, Laikova et al. developed spring wheat cultivars with superior agronomic performance through the incorporation of *A. tauschii* genetic material [109].

**Figure 4:**
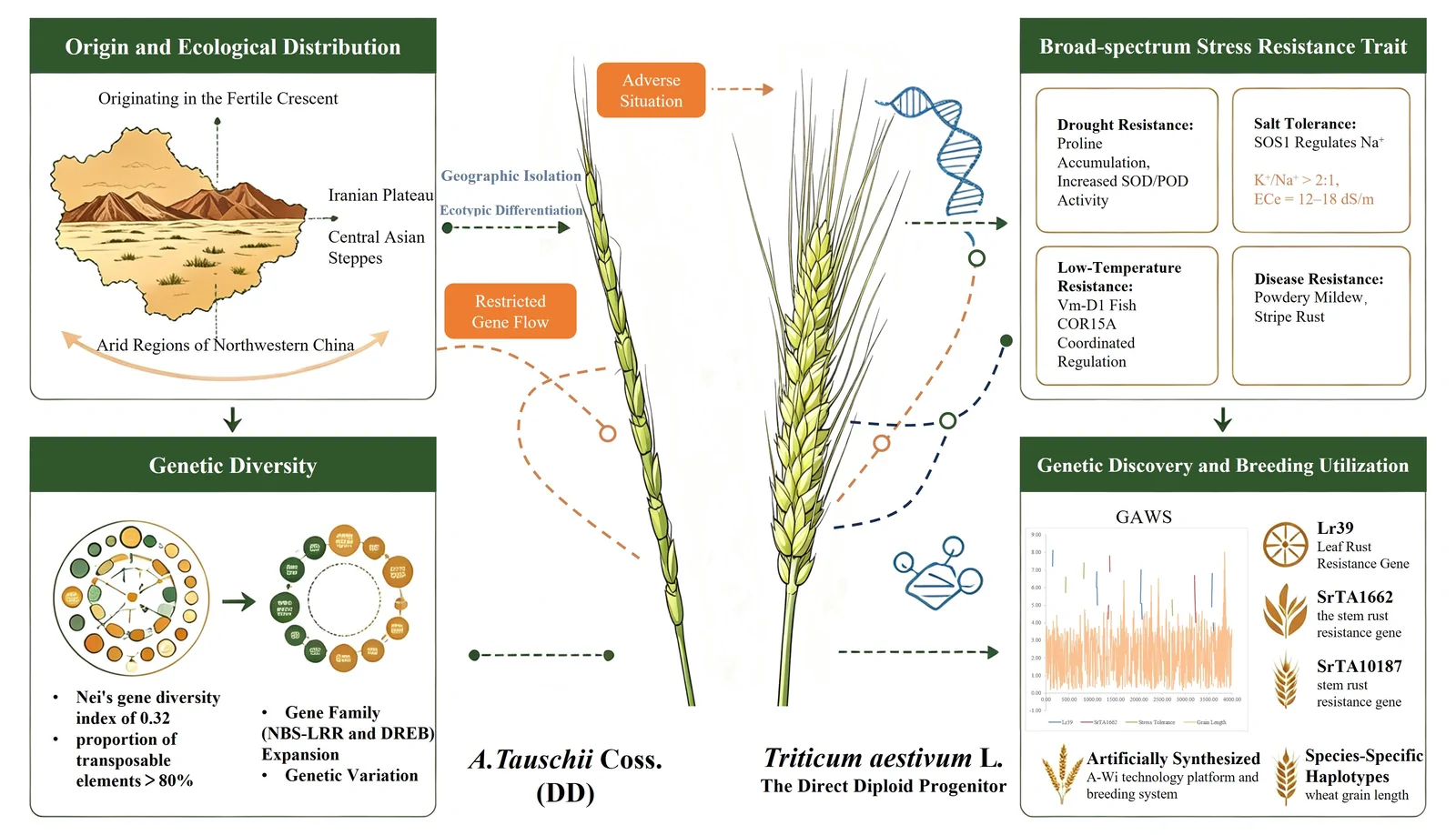
Genetic Resources and Gene Discovery.

Guided by the breeding objectives of high yield, superior grain quality, stress tolerance, yield stability, and adaptation to climate change, Professor Song Chunpeng’s research group established a strategy for the targeted introgression of elite genes from wild relatives contributing to the wheat D subgenome. They subsequently developed the *A. tauschii*-wheat introgression (A-Wi) technology platform and breeding system. This platform enables the simultaneous improvement of disease resistance, grain quality, and yield-related traits while providing a broadly applicable technical framework for the genetic enhancement of cereal crops [108]. Overall, as a natural reservoir of genetic diversity for common wheat [108,109,110], *A. tauschii* has irreplaceable value for elucidating the evolutionary history of wheat polyploidization, expanding the genetic diversity of cultivated wheat, and developing next-generation elite cultivars (Figure 4). Future research should prioritize the systematic collection and precise characterization of *A. tauschii* germplasm, comprehensive mining of elite allelic variation, and the integration of emerging technologies, including genomics, gene editing, and synthetic biology. These efforts will accelerate the identification, introgression, and pyramiding of favorable genes, thereby providing a robust genetic foundation for wheat germplasm innovation and sustainable breeding.

## 6. Conclusions and Prospects

*A. tauschii* holds unique dual significance in wheat production systems: as a highly competitive invasive weed, it threatens yield stability across major wheat-growing regions, while as the D-genome progenitor of common wheat, it provides an invaluable reservoir of genetic diversity for crop improvement. This review systematically synthesizes current advances in the taxonomy, population differentiation, invasion ecology, damage mechanisms, integrated management, and genetic resource utilization of *A. tauschii*, yielding several key recognitions.

First, weedy *A. tauschii* populations in China have evolved strong adaptive alignment with winter wheat agroecosystems, including highly synchronized seedling emergence timing, broad environmental tolerance, and prolific reproductive capacity. These traits, combined with predominantly seed-mediated dispersal via contaminated commercial seed lots and cross-regional agricultural machinery, underpin its rapid spread and severe competitive suppression of wheat growth. Second, sustainable and durable control of *A. tauschii* cannot be achieved through any single measure. An integrated framework combining agronomic regulation, quarantine-based dispersal interruption, judicious chemical intervention, and smart agricultural monitoring delivers the most reliable long-term efficacy, as validated by regional field practices. Third, the rich genetic diversity harbored by wild and weedy *A. tauschii* populations—particularly for abiotic stress tolerance and broad-spectrum disease resistance—offers enormous untapped potential for wheat genetic improvement, and established whole-genome introgression platforms have greatly lowered the barrier to its efficient utilization in breeding.

Looking forward, three key research priorities should be highlighted to advance the field. First, a nationwide monitoring and early-warning network integrating remote sensing, ecological niche modeling, and molecular detection should be established to track population dynamics, range expansion, and herbicide resistance evolution in real time. Second, the molecular mechanisms underlying *A. tauschii* invasiveness and stress adaptation should be deeply dissected; such research will not only inform the development of targeted weed management technologies but also accelerate the mining and functional verification of elite alleles for wheat breeding. Third, environmentally friendly and precision-oriented integrated management systems should be further optimized, with emphasis on reducing herbicide reliance, developing novel biological control approaches, and balancing weed suppression with in situ conservation of valuable *A. tauschii* germplasm.

Through interdisciplinary integration of weed science, ecology, molecular genetics, genomics, and digital agriculture, *A. tauschii* management will shift from passive reactive control to proactive prevention and ecological regulation, ultimately realizing the dual goals of effective weed management and efficient utilization of genetic resources for sustainable wheat production.

## Figures and Tables

**Table 1:** Ecological Adaptation of *A. tauschii* to Environmental Factors.

Test Material/Population	Environmental Factor	Treatment Gradient/Level	Main Results	References
*A. tauschii* seeds were collected from winter wheat fields in Jining, Shandong, Zhumadian, Henan and Bangbu, Anhui.	Temperature	Set 8 constant temperature treatments at 5, 10, 15, 20, 25, 30, 35 and 40 °C.	The optimum germination temperature of *A. tauschii* is 15~20 °C.	[54]
	pH	Prepare buffers with pH of 3.0, 4.0, 5.0, 6.0, 7.0, 8.0, 9.0 and 10.0 respectively.	The seeds of *A. tauschii* can germinate in buffer with pH 3~10, and the germination rate is above 82%.	
	Osmotic potential	Prepare water solutions with water potentials of 0, −0.1, −0.3, −0.5, −0.7, −0.9, −1.1, −1.3, −1.5 and −1.7 MPa respectively.	With the decrease in water potential, the germination rate of *A. tauschii* was positively correlated with the water potential.	
	Salt stress	Prepare sodium chloride solutions with concentrations of 0.0, 20.0, 40.0, 80.0, 120.0, 160.0, 200.0, 240.0, 280.0, 320.0, 360.0 and 400.0 mM respectively.	The germination rate of *A. tauschii* was negatively correlated with salt concentration.	
*A. tauschii* seeds came from the experimental field of Xinxiang Academy of Agricultural Sciences, Henan Province.	Salt stress	NaCl and Na_2_CO_3_ solutions with different concentrations [0(CK), 50, 100, 150 and 200 mmol/L].	With the increase in the concentration of two kinds of salt treatment solutions, the germination rate of *A. tauschii* seeds decreased continuously.	[55]
Tausch’s goatgrass population were collected from a winter wheat field in Yongnian of Hebei Province (36°45′ N, 114°29′ E), China.	Temperature	Ten temperature regimes, 0, 5, 10, 15, 20, 25, 30, 35, 40 and 45 °C, were employed to determine the optimum temperature for seed germination.	Tausch’s goatgrass seed germinated from a wide range of temperature (5~35 °C); the optimum temperature was between 15~25 °C, with 92–97% germination.	[56]
	pH	The effect of pH on germination was studied using buffer solutions with pH values ranging from 3.0 to 10.0 prepared as described by Chachalis and Reddy.	Tausch’s goatgrass seed germination was greater than 92% over a pH range of 3.0 to 10.0.	
	Osmotic potential	Aqueous solutions with osmotic potential of 0, −0.1, −0.3, −0.5, −0.7, −0.9, −1.1, −1.3, −1.5, and −1.7 Mpa.	Tausch’s goatgrass germination was 98, 96, and 70% at osmotic potentials of 0, −0.7, and −1.7 Mpa.	
	Salt stress	Placing the seeds in Petri dishes containing different concentrations (0, 20, 40, 80, 120, 160,200, 240, 280, 320, 360, and 400 mM) of sodium chloride solutions.	Germination of Tausch’s goatgrass seeds inversely related to NaCl concentration.	
*A. tauschii* seeds were collected from Xiangcheng, Henan Province, China. Winter wheat fields in Liaocheng City, Shandong Province and Weinan City, Shaanxi Province	pH	Set the PH of the buffer solution to 3–10.	There was no significant difference in the germination rate of *A. tauschii* in the range of pH 3−10.	[57]
	Osmotic potential	The water permeability is 0.0, −0.1, −0.3, −0.5, −0.7, −1.0 and −1.5 MPa.	*A. tauschii* seeds are sensitive to high water potential, and their germination rate decreases with the decrease in water potential.	
	Salt stress	0, 20, 60, 120, 180, 240, 300, 360, 420, 480 mmol/L NaCl solution.	*A. tauschii* seeds are relatively salt-tolerant.	
*A. tauschii* seeds were taken from Xinxiang Academy of Agricultural Sciences experimental site, which is the mature seed of that year.	Temperature	Set three temperature gradients of 15 °C, 20 °C and 25 °C.	With the increase in temperature, the emergence rate of *A. tauschii* increased first and then decreased.	[61]
Mature *A. tauschii* seeds	pH	The pH value is 4, 5, 6, 7, 8, 9 and 10.	The pH range required for seed germination of *A. tauschii* is wide.	[62]
	Osmotic potential	The osmotic potential is −0.1, −0.2, −0.3, −0.4, −0.5 and −0.6 MPa.	The seed germination of *A. tauschii* has a moderate level of salt tolerance, and the osmotic potential of −0.4~−0.1 Mpa can significantly promote the seed germination and germination of *A. tauschii*.	
	Salt stress	20, 40, 80, 160 mmol/L NaCl solution and Na_2_SO_4_ solution.	With increasing salt concentration, the germination rate and germination rate of *A. tauschii* seeds decreased gradually.	

## Data Availability

No new data were created or analyzed in this study. Data sharing does not apply to this article.
